# Prognostic factors of BRAF V600E colorectal cancer with liver metastases: a retrospective multicentric study

**DOI:** 10.1186/s12957-022-02594-2

**Published:** 2022-04-23

**Authors:** Sahir Javed, Stéphane Benoist, Patrick Devos, Stéphanie Truant, Rosine Guimbaud, Astrid Lièvre, David Sefrioui, Romain Cohen, Pascal Artru, Aurélien Dupré, Jean-Baptiste Bachet, Christelle de la Fouchardière, Anne Ploquin, Anthony Turpin

**Affiliations:** 1 Department of Medical Oncology, Centre Oscar Lambret, F-59000 Lille, France; 2 Department of Medical Oncology, Centre Hospitalier de Valenciennes, F-59300 Valenciennes, France; 3 Department of Digestive Surgery and Surgical Oncology, Bicêtre Hospital, AP-HP, Paris–Sud University, Paris, France; 4 University of Lille, CHU Lille, F-59000 Lille, France; 5 Department of Digestive Surgery and Transplantation, CHU Lille, F-59000 Lille, France; 6 Department of Medical Oncology, Claudius Regaud Institute, Toulouse, France; 7 Department of Gastroenterology, CHU Pontchaillou, Rennes 1 University, Rennes, France; 8 Digestive Oncology Unit, Department of Hepatogastroenterology, Rouen University Hospital, Normandie Université, UNIROUEN, Inserm 1245, IRON group, Rouen, France; 9 Medical Oncology Department, Saint-Antoine Hospital, Sorbonne University, AP-HP, F-75012 Paris, France; 10 Department of Gastroenterology, Jean Mermoz Hospital, Lyon, France; 11 Department of Surgical Oncology, Leon Berard Cancer Center, UNICANCER, Lyon, France; 12 Department of Hepato-Gastroenterology, Hôpital Pitié Salpêtrière, Assistance Publique – Hôpitaux de Paris (AP-HP), Paris, France; 13 Medical Oncology Department, Centre Leon Berard, Lyon I University, Lyon, France; 14 Department of Oncology, CHU Lille, F-59000 Lille, France; 15 Univ. Lille, CNRS, Inserm, CHU Lille, UMR9020-U1277 - CANTHER - Cancer Heterogeneity Plasticity and Resistance to Therapies, F-59000 Lille, France

**Keywords:** Colorectal cancer, *BRAF* mutation, Drug therapy, Liver metastasis surgery

## Abstract

**Background:**

*BRAF* V600E-mutant colorectal cancers (CRCs) are associated with shorter survival than *BRAF* wild-type tumors. Therapeutic decision-making for colorectal liver metastases (CRLM) harboring this mutation remains difficult due to the scarce literature. The aim was to study a large cohort of *BRAF* V600E-mutant CRLM patients in order to see if surgery extend overall survival among others prognostic factors.

**Methods:**

*BRAF* V600E-mutant CRCs diagnosed with liver-only metastases, resected or not, were retrospectively identified between April 2008 and December 2017, in 25 French centers. Clinical, molecular, pathological characteristics and treatment features were collected. Overall survival (OS) was defined as the time from CRLM diagnosis to death from any cause. Cox proportional hazard models were used for statistical analysis.

**Results:**

Among the 105 patients included, 79 (75%) received chemotherapy, 18 (17%) underwent upfront CRLM surgery, and 8 (8%) received exclusive best supportive care. CRLM surgery was performed in 49 (46.7%) patients. CRLM were mainly synchronous (90%) with bilobar presentation (61%). The median OS was 34 months (range, 28.9–67.3 months) for resected patients and 10.6 (6.7–12.5) months for unresected patients (*P* < 0.0001). In multivariate analysis, primary tumor surgery (hazard ratio (HR) = 0.349; 95% confidence interval (CI) 0.164–0.744, *P* = 0.0064) and CRLM resection (HR = 0.169; 95% CI 0.082–0.348, *P* < 0.0001) were associated with significantly better OS.

**Conclusions:**

In the era of systemic cytotoxic chemotherapies, liver surgery seems to extend OS in BRAF V600E-mutant CRCs with liver only metastases historical cohort.

## Background

Approximately 50% of patients with colorectal cancer develop colorectal liver metastases (CRLM), and their outcomes are intimately related to CRLM resectability: the 5-year overall survival (OS) rate ranges from 30 to 50% after CRLM surgery, whereas it is lower than 10% for unresectable CRLM [1, 2]. However, 50 to 85% of patients experience relapse after CRLM resection, and the curative intent of metastasectomy is accomplished in approximately 20% of cases [3–5]. In the era of precision medicine, efforts are aimed at a better selection of patients who might benefit from metastasectomy. Several clinical scoring systems based on clinicopathological parameters have been proposed; but their clinical value is still questioned [6, 7].

Colorectal cancers (CRCs) harboring *BRAF* V600E mutations are aggressive cancers with rapid metastatic spread that more frequently involves peritoneal and nodal invasion than liver metastases. Until recently, their management was based on limited data, mainly from subgroup analysis of randomized clinical trials. This subgroup of patients is less responsive to standard chemotherapies. In the CALGB/SWOG 80405 trial assessing the addition of the targeted agent cetuximab or bevacizumab or both to doublet chemotherapy FOLFOX or FOLFIRI, the median OS for *BRAF*-mutant patients remained poor compared to that of *BRAF* wild-type patients: 13.5 months versus 30.6 months, respectively [8]. In addition, a recent meta-analysis of five randomized clinical trials demonstrated that intensive upfront chemotherapy with triplet FOLFOXIRI plus bevacizumab did not improve survival among *BRAF* V600E-mutant patients [9].

This gap in survival rates has been also observed after CRLM resection in several retrospective subgroup analyses. In the latest study, the 3-year OS rates for *BRAF*-mutant and wild-type patients were 54% and 82.9%, respectively [10]. However, these numbers must be interpreted with caution as *BRAF*-mutant CRCs with liver-only metastases represent a limited population, and only 5% of patients undergoing CRLM resection harbor these mutations [11–13].

Therefore, our knowledge about *BRAF*-mutant patients with CRLM is currently limited to those patients undergoing resection or with extra-hepatic metastases receiving chemotherapies. The aim of the study was to report outcomes of a large cohort of *BRAF* V600E-mutant patients with exclusive CRLM and to identify if surgery is a prognostic factor among others.

## Methods

### Study population and design

Data from 105 patients diagnosed with liver-limited CRC metastases harboring *BRAF* V600E mutations between April 1, 2008, and December 31, 2017, were retrospectively collected from 25 French hospitals. Exclusion criteria were as follows: presence of extra-hepatic metastases, date of CRLM diagnosis not available, and follow-up less than 12 months. Data from the majority of patients who underwent CRLM resection came from databases of the following four French scientific groups: Fédération de Recherche et Chirurgie (FRENCH), Association de Chirurgie Hépato-Bilio-Pancréatique et Transplantation (ACHBT), Association des Gastro-Enterologues Oncologues (AGEO), and the PRODIGE group (Partenariat de Recherche en Oncologie DIGEstive). *BRAF* V600E mutated-patients were identified from molecular biology platforms and each case was screened in order to identify and include patients with liver-only disease. Patients with synchronous extra-hepatic resectable disease metastases were excluded. The study was conducted according to the ethical standards in line with the French regulation. French Data Protection Authority (CNIL agreement n° DEC18-409 (2018_01)) provided a waiver of informed consent for this retrospective study and permitted the publication of anonymized data.

*BRAF* and *RAS* mutational statuses were determined from either primary CRC samples or CRLM tissues—as several studies have demonstrated a high molecular concordance between primary CRC and liver metastases [14, 15]—using PCR or next-generation sequencing according to the technology available at each hospital.

The following clinical, molecular, and pathological characteristics were collected at baseline: age at CRLM diagnosis, sex, *KRAS* and *NRAS* mutations, mismatch repair (MMR) status, primary tumor site, surgery of primary tumor, tumor and nodal stages according to the American Joint Committee on Cancer (AJCC), synchronous (<6 months) versus metachronous CRLM diagnosis, CRLM distribution and number, and initial resectability status. In addition, treatment features (CRLM surgery and systemic therapies) and survival were assembled.

MMR status was assessed by both immunohistochemical analysis of microsatellite instability-high (MSI-H) defined by loss of MLH1, MSH2, MSH6, or PMS2 expression and/or PCR. Right-sided tumors were defined as arising from the caecum to the transverse colon and left-sided tumors as arising from the splenic flexure to the rectosigmoid junction.

### Treatment features and definitions

The treatment decision for each patient was made during multidisciplinary meetings in each institution. According to the CRLM resectability status and performance status, patients received preoperative chemotherapy, upfront liver surgery, palliative chemotherapy, or best supportive care. Patients were then followed-up every 2–3 months through physical examination, biological tests, and computed tomography scan. OS was defined as the time from CRLM diagnosis to the time of death or the date of last follow-up. Postoperative OS was defined as the time from CRLM resection. OS rate was determined at December 31, 2018.

### Statistical analysis

For descriptive analysis, quantitative parameters are presented as median and quartiles and qualitative parameters as percentages. CRLM resected and unresected groups were compared using the *χ*^2^ or Fisher’s exact test, as appropriate. Survival rates were estimated by the Kaplan-Meier method and were compared using the log-rank test. After univariate analysis, variables with *p* < 0.2 and with less than 33% missing data were integrated in a backward selection for final multivariate Cox model. Boostrap methods were also used. The variables of interest were as follows: age, gender, primary tumor site, primary tumor surgery, synchronous CRLM, CRLM number, CRLM distribution, resectability status, metastasectomy, and the use of first-line chemotherapy and targeted therapies. All reported *p* values are two-sided, and *P* < 0.05 was considered statistically significant. Statistical analyses were performed using SAS V9.4 (Cary, NC, USA).

## Results

### Patient characteristics

One-hundred and five patients were identified with *BRAF* V600E-mutant CRLM diagnosed between April 2008 and December 2017. The median age at CRLM diagnosis was 67 years. CRLM were mainly synchronous (90%) with bilobar presentation (61%). One patient harbored co-*KRAS* mutation. MMR status was available for 69 patients (66%): 21 patients (30%) were identified with an MSI-H phenotype. Clinical, molecular, and pathological characteristics are summarized in Table 1.

**Table 1:** Patient characteristics according to CRLM status (resected or unresected)

Variables	Total (*n* = 105)	Resected CRLM (*n* = 49)	Unresected CRLM (*n* = 56)	*P* value
Gender				
Male	51 (49%)	20 (41%)	31 (55%)	0.1369
Female	54 (51%)	29 (59%)	25 (45%)	
Age				
**≤**65 years	42 (40%)	20 (41%)	22 (39%)	0.8731
**>**65 years	63 (60%)	29 (59%)	34 (61%)	
Primary tumor site				
Right-sided	56 (55%)	26 (54%)	30 (56%)	0.0911
Left-sided	28 (27%)	17 (36%)	11 (20%)	
Rectum	18 (18%)	5 (10%)	13 (24%)	
Missing data	3	1	2	
Primary tumor surgery				
Yes	79 (75%)	49 (100%)	30 (54%)	< 0.0001
No	26 (25%)	0	26 (46%)	
T stage				
T1–T2	4 (5%)	4 (8%)	0	0.2909
T3–T4	73 (95%)	44 (92%)	29 (100%)	
Missing data	28	1	27	
N stage				
N0	9 (12%)	7 (15%)	2 (7%)	0.4699
N1–N2	68 (88%)	41 (85%)	27 (93%)	
Missing data	28	1	27	
CRLM time of diagnosis				
Synchronous	94 (90%)	41 (84%)	53 (95%)	0.0671
Metachronous	11 (10%)	8 (16%)	3 (5%)	
CRLM distribution				
Unilobar	33 (39%)	21 (57%)	12 (26%)	0.0036
Bilobar	51 (61%)	16 (43%)	35 (74%)	
Missing data	21	12	9	
Number of CRLM				
<10	56 (70%)	32 (89%)	24 (55%)	< 0.0001
**≥**10	24 (30%)	4 (11%)	20 (45%)	
Missing data	25	13	12	
Initial resectability				
Yes	43 (52%)	32 (86%)	11 (24%)	< 0.0001
No	39 (48%)	5 (14%)	34 (76%)	
Missing data	23	12	11	
*RAS* status				
Wild-type	103 (99%)	47 (98%)	56 (100%)	0.4615
Mutant	1 (1%)	1 (2%)	0	
Missing data	1	1	-	
MSI-H				
Yes	21 (30%)	14 (29%)	7 (33%)	0.7293
No	48 (70%)	34 (71%)	14 (67%)	
Missing data	36	1	35	
Resection margin status				
R0	32 (89%)	32 (89%)	NC	
R1 parenchymal	4 (11%)	4 (11%)	NC	
R1 vascular	0	0	NC	
Missing data	13	13		

*CRLM* Colorectal liver metastases, *MSI-H* Microsatellite instability. *χ*^2^ or Fisher’s exact test was applied for groups comparisons. *NC* Not concerned

### Treatment features

**Fig. 1:**
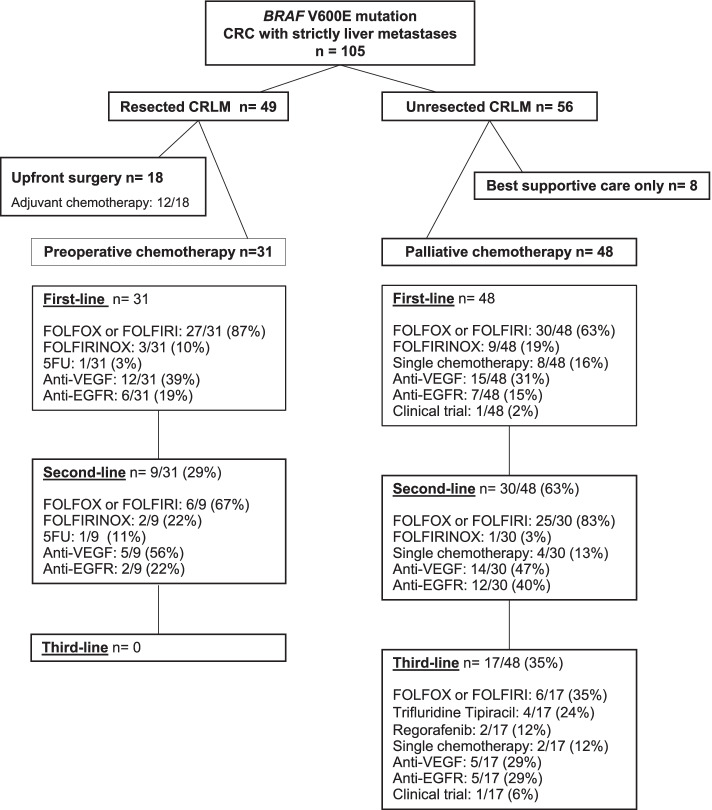
Flow chart of included patients (*n*= 105)

The flow chart in Fig. 1 describes the treatments administrated. Forty-nine out of 105 patients (47%) underwent CRLM resection, of which 31 (63%) after chemotherapy. The details about surgical procedures were available for 37 out of 49 patients: major liver resection (≥3 segments) was performed in 38% of cases (14/37), two-stage liver resection in 24% of cases (9/37), and preoperative portal vein embolization in 11% of cases (4/37). Radiofrequency ablation was combined with liver surgery in 24% of patients (9/37). R1 parenchymal resections were present in 4 out of 36 cases (11%). Sixty-five percent received adjuvant chemotherapy.

Cytotoxic doublet chemotherapies (FOLFOX or FOLFIRI) represented the main first-line treatment (72%, *n*=57/79), followed by triplet chemotherapy (FOLFIRINOX) for 12 patients (15%). Twenty-seven patients (34%) received bevacizumab in combination with chemotherapy as the first-line treatment.

Among patients treated exclusively with chemotherapy (*n* = 48), 63% received a second line (*n* = 30) and 35% received a third line (*n* = 17). From the second line onward, targeted therapies were more frequently used. In total, 24 patients (50%) received concomitantly or successively the following cytotoxic drugs: fluoropyrimidine, oxaliplatin, and irinotecan. Of note, 54% of patients with unresected CRLM underwent primary tumor surgery (Table 1).

Finally, 10 out of 105 patients (10%) participated in clinical trials, four of which involved immune checkpoint inhibitors (ICIs) or targeted therapies.

Table 2 summarizes the results of descriptive and univariate survival analysis according to treatments.

**Table 2:** Descriptive and univariate survival analysis according to treatment feature

Treatment characteristic	Median OS (months)	***P*** value
CRLM surgery		
Yes	34	*P* < 0.0001
No	10.6	
Upfront CRLM surgery		
Yes	33	*P* = 0.3402
No	34	
Postoperative OS	28	NC
Exclusive chemotherapy	11.5	NC
FOLFIRINOX +/− bevacizumab	16.6	NC

*CRLM* Colorectal liver metastases, *OS* Overall survival, *NC* Not concerned

### Prognostic factors

**Fig. 2:**
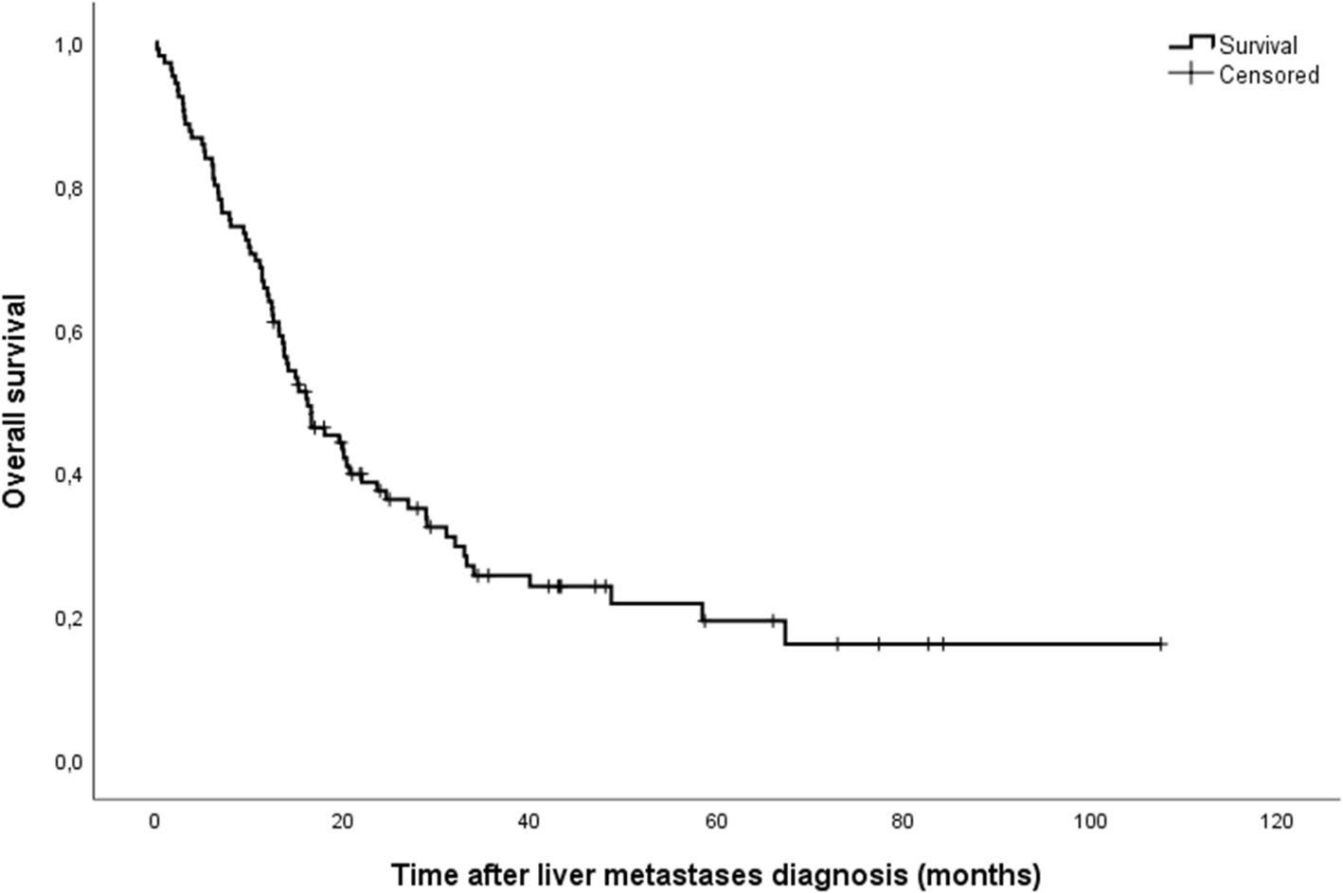
Overall survival for *BRAF*-mutant colorectal cancer with exclusive CRLM (*n* = 105), median OS = 16.2 months

The median OS was 16.2 months (95% confidence interval (CI): 13.2–20.7), with a 1-year OS rate of 65% and a 3-year rate of 16% (Fig. 2).

In univariate analysis, the following factors were associated with longer survival: CRLM resection, primary tumor surgery, CRLM less < 10, and initially resectable CRLM (Table 3).

**Table 3:** Univariate and multivariate Cox analysis of prognostic factors for OS (*n* = 70). All other parameters (sex, age, etc.) are not significant at *P* = 0.20

	Univariate analysis		Multivariate analysis	
	HR [95% CI]	*P* value	HR [95% CI]	*P* value
CRLM resection	0.131 [0.068–0.253]	<0.0001	0.169 [0.082–0.348]	<0.0001
Primary tumor resection	0.135 [0.065–0.280]	<0.0001	0.349 [0.164–0.744]	0.0064
CRLM ≥10	3.612 [1.973–6.613]	<0.0001	-	NS
Non-resectable CRLM	2.931 [1.632–5.262]	0.0003	-	NS
Bilobar CRLM	1.697 [0.931–3.092]	0.0842	-	NS
Rectum primary site	1.792 [0.902–3.561]	0.0957	-	NS

*CRLM* Colorectal liver metastases, *NS* Not significant

In multivariate analysis, primary tumor surgery (hazard ratio (HR) = 0.349; 95% CI 0.164–0.744, *P* = 0.0064) and CRLM resection (HR = 0.169; 95% CI 0.082–0.348, *P* < 0.0001) were associated with significantly better OS.

**Fig. 3:**
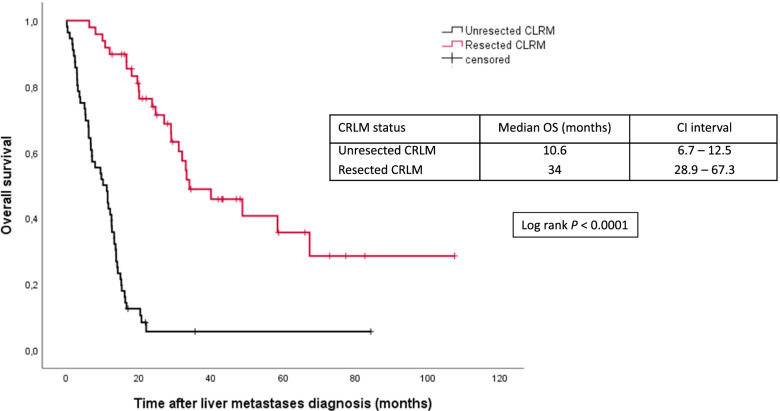
Overall survival according to CRLM resection status. CRLM, colorectal liver metastases; OS, overall survival; CI, confidence interval

CRLM resection was associated with a significantly longer OS, with a median of 34 months (range, 28.9–67.3 months) versus 10.6 (6.7–12.5) months for unresected patients, *P* < 0.0001 (Fig. 3). Patients who received preoperative chemotherapy had a median OS of 34 months (28.9–non-evaluable) versus 33 months (19.6–non-evaluable) for patients resected upfront (*P* = 0.3402). The median postoperative OS was 28 (19.8–53.5) months.

**Fig. 4:**
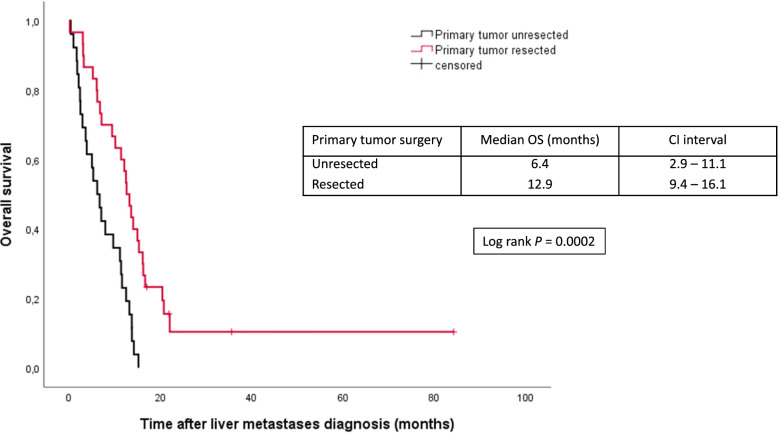
Overall survival according to primary tumor resection status among unresected CRLM group (*n* = 56). OS, overall survival; CI, confidence interval

Primary tumor surgery was associated with a significantly longer OS, with a median of 23.7 (16.6–33) months versus 6.4 (2.9–11.1) months for unresected patients (*P* < 0.0001). The benefit of primary tumor surgery remained statistically significant in the unresected CRLM group (*n* = 30), with a median OS of 12.9 (9.4–16.1) months versus 6.4 (2.9–11.1) months in the unresected group (primary tumor and CRLM, *n* = 26) (*P* = 0.0002) (Fig. 4).

Exclusive chemotherapy treatment conferred a median OS of 11.5 months (7.1–13.2).

### Comparison of resected and non-resected CRLM groups

Clinical and pathological characteristics according to CRLM treatment status are summarized in Table 1. Patients with resected CRLM (*n* = 49) were significantly more likely to present less than 10 metastases (*P* < 0.0001) with unilobar distribution (*P* = 0.0036) and initially resectable (*P* < 0.0001). Considering the missing data about MSI-H status, no significant difference between the two groups was observed (*P* = 0.7293). Concerning the radiologic responses, among the patients treated exclusively with chemotherapy, 29% (14/48) had an objective response, 27% (13/48) had stable disease, and 44% (21/48) were progressive. Data were available for 25 patients who received preoperative chemotherapy, with a stable disease rate of 28% (7/25), objective response rate of 64% (16/25), and progressive disease rate of 8% (2/25).

### Sites of progression

In total, 95 out of 105 patients (90%) experienced disease relapse after liver surgery (*n* = 40) or disease progression (*n* = 55) by the end of follow-up. The liver was the main site of disease recurrences after liver surgery or progression after chemotherapy. Patients with unresected CRLM experienced more peritoneal progression than patients with CRLM resected (17.5% versus 7%).

## Discussion

Data on patients with *BRAF* V600E-mutant CRC and liver-only metastases are scarce. Most of the published studies on BRAF mutated-CRLM included only patients who underwent surgery. This was the largest dedicated cohort (*n* = 105, regardless of treatments received) study to date, bringing an additional support that their resection is beneficial vs chemotherapy only.

The profit of liver resection is in line with findings in two recent retrospective studies. Johnson et al. showed that among 52 patients with *BRAF* V600E metastatic CRC, the median OS was significantly prolonged when liver resection with curative intent was performed: 29.1 versus 22.7 months, HR 0.33, 95% CI: 0.12–0.78, *P* = 0.01 [16]. In the second study, 43 out of 282 patients underwent surgery, with a median OS of 47.4 months versus 19.5 months for those who had no metastasectomy (HR 0.469, 95% CI: 0.288–0.765; *P* = 0.0024) [17]. In addition, a recent case-matched study demonstrated that *BRAF* mutations did not increase the risk of relapse after CRLM surgery compared to *BRAF* wild-type disease (HR 1.16, 95% CI 0.72–1.85; *P* = 0.547) [10]. The high proportion of patients undergoing resection in our cohort should reflect the fact that the assessment of mutational status was probably not performed in patients with poor prognosis.

To allow comparison with unresected patients, we defined OS as the time from CRLM diagnosis, whereas previous studies have reported OS from the date of liver surgery. Nonetheless, the median post-operative OS starting from the date of surgery in this cohort (28 months) was in line with those in previous studies: from 22.6 months reported by Schirripa et al. (*n* = 12) to 47.4 months reported by De la Fouchardière et al. (*n* = 35) [17–20]. OS results remain lower than the previous publication from Bachet et al. with a median OS of 52.7 months (*n*= 66). The exclusion of non-V600E *BRAF* mutated-patients in our study may explain this difference [10]. Relapse-free survival, progression-free survival, and disease-specific survival were not included in the present study as the definitions would differ for resected and unresected groups.

The positive results of primary tumor resection in the unresected CRLM group were surprising and should be interpreted with caution due to the small number of patients involved (30 versus 26). Indeed, a recent study showed that primary tumor resection followed by chemotherapy was not superior to chemotherapy alone (HR 1.10 [0.76–1.59], one-sided *P* = 0.69). The trial was terminated early for futility reason [21].

In this cohort, a small proportion of patients received an upfront triplet regimen with or without bevacizumab (12/79, 15%), with a median OS of 16.6 months (6.7–not reached). Of note, the majority of the cohort presented a good performance status (84% in the group treated exclusively with chemotherapy). An intensive regimen has been assumed beneficial in *BRAF*-mutant patients with unresectable liver-limited disease to date, based on a pooled analysis of a small number of patients (*n* = 20) [22]. However, a recent meta-analysis of five randomized trials comparing FOLFOXIRI plus bevacizumab with doublet plus bevacizumab in 105 *BRAF* V600E-mutant patients showed no increased benefit in the intensive therapeutic arm [9].

Among the patients treated with chemotherapy only, 63% received second-line treatment; this rate is superior to those reported in the COIN trial (33%) [23] and in a matched case-control study (51%) [24]. Beyond the second line, candidates for treatment decreased dramatically to 35%, and it is important to pinpoint that only 50% of patients received the three major cytotoxic drugs: 5-FU/leucovorin, oxaliplatin, and irinotecan. The present study showed that even if *BRAF*-mutant metastatic disease is confined to one organ, the prognosis remains poor when the patient is treated with chemotherapy only.

The study population mostly received standard chemotherapies, not new practice-changing therapies. Recently, mitogen-activated protein kinase pathway-targeted therapies have demonstrated better efficacy. In the BEACON trial, the combination of encorafenib, a BRAF inhibitor, and cetuximab (anti-EGFR) with or without binimetinib, a MEK inhibitor, was associated with a significantly longer OS than standard chemotherapy after at least one prior line in a large cohort of patients with *BRAF* V600E mutations [25]. The same regimen as a first-line is currently under investigation in a phase II trial (ANCHOR-CRC) [26].

ICIs represent another therapeutic option, especially for MSI-H mCRC. The phase III KEYNOTE-177 study demonstrated that first-line pembrolizumab was associated with significant progression-free survival improvement over chemotherapy in MSI-H mCRC (median progression-free survival of 16.5 versus 8.2 months, HR = 0.60; 95% CI 0.45–0.80, *P* = 0.0002). The benefit of pembrolizumab was consistent in the *BRAF* V600E-mutant subgroup [27]. In the present cohort, very few patients received ICI after chemotherapy failure (*n* = 3), explained by the period of inclusion. MSI-H status was not reliable for any conclusions due to insufficient data. In a recent study, MSI-H status was associated with significantly longer OS in a *BRAF*-mutant mCRC population (*n* = 194) treated with standard chemotherapies [17]. In the era of immunotherapy, the impact of immune cell infiltrations in *BRAF*-mutated colorectal cancers is questioned [28, 29].

The major weakness of the study is related to the differences between the resected and unresected CRLM groups with the bias of less aggressive disease in the resected group. However, this should be counterbalanced by increased liver surgery ability, and moreover, the initial resectability or unresectability status might have been subject to variability between the centers. The reasons for non-resectability were not specified, and patients could be considered unresectable solely based on the presence of *BRAF* mutations.

The missing data in this cohort represent an important limitation, and some known prognostic factors, such as MSI status, were not included in the statistical analysis. Therefore, a case-matched study (resected and unresected CRLM) was not feasible. A prospective study with current therapeutic strategies (ICI for MSI-H and anti-BRAF plus anti-MEK for non-MSI-H) should be considered.

With all the limitations of a retrospective study, this was conducted in the largest cohort of *BRAF* V600E mutant patients with CRLM reported to date. This will be considered therefore as a historical cohort of BRAF mutated patients who had a liver surgery before the advent of immunotherapies and combinations of anti-BRAF, anti-MEK, and anti-EGFR targeted therapies. A subgroup difficult to look at given its rarity, also prospective studies would be difficult to realize.

## Conclusions

In BRAF V600E mutated patients, as long as systemic targeted therapies and immunotherapies are under development, liver resection is, with primary tumor resection, the only prognosis factor for overall survival. While our population is heterogeneous because of the lack of data about MMR phenotype, we show that those patients should not be excluded from liver surgery.

## Data Availability

The datasets used and/or analyzed during the current study are available from the corresponding author on reasonable request.

## Abbreviations

CI: Confidence interval
CRC: Colorectal cancer
CRLM: Colorectal liver metastases
HR: Hazard ratio
ICI: Immune checkpoint inhibitor
MMR: Mismatch repair
MSI-H: Microsatellite instability
OS: Overall survival
PCR: Polymerase chain reaction
